# IMPLEMENTATION OF PATIENT PRIORITIES CARE WITHIN A VA GERIATRICS CLINIC

**DOI:** 10.1093/geroni/igz038.2844

**Published:** 2019-11-08

**Authors:** Jennifer Freytag, Lilian Dindo, Angela Catic, Aanand Naik, Mary Tinetti

**Affiliations:** 1 Center for Innovations in Quality, Effectiveness and Safety, Houston, Texas, United States; 2 Michael E DeBakey VA Medical Center, Houston, Texas, United States; 3 Yale University, New Haven, Connecticut, United States

## Abstract

Patient Priorities Care (PPC) is an approach to decision-making for older adults with multiple chronic conditions (MCC). PPC trains facilitators to have structured conversations with patients to identify their priorities (the outcomes that matter most given what care they are willing/able to do or receive). Clinicians then align care to achieve patient priorities rather than focusing on multiple single-disease guidelines. We piloted PPC in a VA geriatrics clinic and compared it to usual care (UC) for multimorbid adults. This retrospective cohort study (n=36 PPC, 36 UC) describes changes made by clinicians after Veterans with MCC had facilitated conversations in a VA geriatric clinic. UC Veterans were matched by prognosis, same primary clinician, and timeframe. Coders used a standardized rubric to assess documented care within medical records. Changes to care examined include medications added/removed, referrals made/avoided, self-care recommendations, and recommendations for social engagement. Although PPC and UC patients were seen by the same clinicians, patients receiving PPC had fewer added medications (mean difference -.47, t(70)=-1.99, p=.05); received more recommendations for self-care aligned with priorities (mean difference .25, t(69)=2.14, p=.003); received more recommended consultations with desired care, including podiatry, transportation, and dermatology (mean difference .55, t(70)=2.06, p=.01), and more recommendations for care and services to facilitate social interactions (p<.0001). PPC produced documented changes in care that better align with patient priorities within the routine care workflow of a busy geriatrics clinic. Our results provide evidence that structured priorities conversations change the way clinicians provide care for older adults with MCC.

